# PD-1/PD-L1 inhibitors combined with chemotherapy or as monotherapy versus chemotherapy alone or observation in triple-negative breast cancer: a systematic review and updated meta-analysis incorporating long-term survival data

**DOI:** 10.3389/fimmu.2026.1958007

**Published:** 2026-10-06

**Authors:** Bing Guan, Yawen Chen, Rong Zhou, Sijia Zhang, Lulu Li, Ping He

**Affiliations:** Department of Pathology, Suzhou BenQ Medical Center, Suzhou, Jiangsu, China

**Keywords:** immune checkpoint inhibitors, meta-analysis, overall survival, pathological complete response, PD-1, PD-L1, pembrolizumab, triple-negative breast cancer

## Abstract

**Background:**

Multiple RCTs have established PD-1/PD-L1 inhibitors as standard in triple-negative breast cancer (TNBC), but prior meta-analyses lacked complete setting coverage, mature long-term survival data, and a quantification of chemotherapy-backbone impact—critical given IMpassion130 (nab-paclitaxel, positive) vs. IMpassion131 (paclitaxel, negative). This update includes final KEYNOTE-522 OS, GeparNuevo 7-year follow-up, and TORCHLIGHT Phase III data, covering neoadjuvant to metastatic disease.

**Methods:**

We searched PubMed and ClinicalTrials.gov to May 2026 plus conference proceedings; an Embase search found no additional trials. Included RCTs compared ICI + chemotherapy vs. chemotherapy alone in TNBC, stratified by setting. The primary endpoints are OS (metastatic) and EFS/iDFS (early-stage). Pooled HRs used random-effects meta-analysis with HKSJ adjustment.

**Results:**

A total of 10 RCTs (5,953 patients) met the inclusion. In the metastatic setting (five RCTs, *n* = 3,526), ICI improved OS (HR 0.85, 95% CI 0.74–0.96; HKSJ-adjusted 0.70–1.03; *I*² = 27%) and PFS (HR 0.82, 0.73–0.92; *I*² = 35%); OS benefit was greater in PD-L1+ patients (HR 0.79, 0.65–0.95; *I*² = 51%). Numerically greater OS with anti-PD-1 vs. anti-PD-L1 was not robust in the sensitivity analysis. In the early-stage setting (five RCTs, *n* = 2,427), ICIs improved EFS/iDFS (HR 0.75, 0.63–0.90; *I*² = 0.2%) and OS (HR 0.64, 0.53–0.78; *I*² = 0%) and increased pCR (OR 1.65, 1.39–1.98; *I*² = 1%). HKSJ adjustment widened the Cis, but the effect patterns remained consistent.

**Conclusions:**

This update confirms a consistent survival benefit of ICI + chemotherapy in both early and metastatic TNBC, which was most pronounced in PD-L1+ populations. Long-term data show durability, especially in neoadjuvant/adjuvant settings. The divergent outcomes with nab-paclitaxel vs. paclitaxel underscore the critical role of the chemotherapy backbone. These findings support current guidelines and highlight the need for refined biomarkers to extend benefit to PD-L1-negative patients.

**Systematic Review Registration:**

https://www.crd.york.ac.uk/prospero/display_record.php?ID=CRD420261356428, identifier CRD420261356428.

## Highlights

- PD-1/PD-L1 plus chemo improves OS (HR 0.85) and PFS (HR 0.82) in metastatic TNBC.
- In early-stage TNBC, ICIs improve EFS (HR 0.75) and OS (HR 0.64).
- Chemotherapy backbone choice critically modulates ICI efficacy.
- Treatment strategy (neoadjuvant + adjuvant vs. neoadjuvant-only vs. adjuvant-only) yields comparable benefit.
- Moderate heterogeneity (*I*² 27%–51%) in metastatic analyses; near-zero heterogeneity (*I*² ~0%) in early-stage analyses.

## 1 Introduction

Triple-negative breast cancer (TNBC), defined by the absence of estrogen receptor, progesterone receptor, and HER2 overexpression, accounts for approximately 15%–20% of all breast cancers and is associated with a disproportionately aggressive clinical course (1, 2). Compared with other breast cancer subtypes, TNBC is characterized by earlier age at diagnosis, higher rates of visceral and central nervous system metastases, and a median overall survival of 12–18 months in the metastatic setting (3, 4). The limited therapeutic armamentarium for this biologically heterogeneous disease has driven an intensive search for effective targeted strategies.

The immunogenic nature of TNBC—evidenced by higher tumor mutational burden, greater stromal tumor-infiltrating lymphocyte (sTIL) density, and elevated PD-L1 expression relative to other breast cancer subtypes—provided a compelling rationale for investigating immune checkpoint inhibitors (ICIs) in this disease (5, 6). Over the past 5 years, a series of landmark randomized controlled trials (RCTs) have evaluated PD-1/PD-L1 inhibitors in combination with standard chemotherapy across both the early-stage and metastatic settings.

The IMpassion130 trial was the first phase III study to demonstrate a progression-free survival (PFS) benefit of atezolizumab (anti-PD-L1) combined with nab-paclitaxel in PD-L1-positive metastatic TNBC (7), leading to accelerated FDA approval in 2019. However, the confirmatory IMpassion131 trial, which substituted solvent-based paclitaxel for nab-paclitaxel, failed to demonstrate either PFS or OS benefit (8), ultimately resulting in the voluntary withdrawal of the atezolizumab indication in the United States. In parallel, KEYNOTE-355 demonstrated a statistically significant and clinically meaningful OS benefit of pembrolizumab (anti-PD-1) combined with chemotherapy in PD-L1 CPS≥10 metastatic TNBC (9), establishing pembrolizumab as the current standard of care in this population.

In the early-stage setting, KEYNOTE-522 demonstrated that neoadjuvant pembrolizumab plus chemotherapy followed by adjuvant pembrolizumab significantly improved both event-free survival (EFS) and, as recently reported, overall survival (10, 11), cementing this regimen as the standard of care for high-risk early-stage TNBC. Additional trials, including IMpassion031 (12), GeparNuevo (13), NeoTRIP (14), and A-BRAVE (15), have further characterized the efficacy of PD-L1 inhibitors in the neoadjuvant and adjuvant settings, yielding a complex evidence landscape with both positive and negative findings.

Several meta-analyses have synthesized the evidence on ICIs in TNBC (16–22). However, these analyses have important limitations. Most were published before the availability of long-term follow-up data from KEYNOTE-522 (final OS analysis, NEJM 2024) and GeparNuevo (7-year follow-up, ESMO 2025). None have comprehensively incorporated the first positive phase III data for toripalimab, a PD-1 inhibitor evaluated in the TORCHLIGHT trial (23), which represents the first Chinese anti-PD-1 antibody to demonstrate OS and PFS benefit in metastatic TNBC. Furthermore, most prior meta-analyses have treated early-stage and metastatic TNBC as a homogenous entity, obscuring differences in treatment effect magnitude and clinical significance between these distinct therapeutic contexts.

We therefore conducted an updated comprehensive systematic review and meta-analysis of all available RCTs comparing PD-1/PD-L1 inhibitors combined with chemotherapy versus chemotherapy alone (or observation) in TNBC. This analysis is distinguished from prior meta-analyses by three features: (1) simultaneous coverage of both early-stage and metastatic settings with mature overall survival data—including the first incorporation of the KEYNOTE-522 final OS analysis (NEJM 2024, median 75-month follow-up) and the 7-year GeparNuevo results (ESMO 2025) into a pooled estimate, (2) inclusion of the first positive phase III data for toripalimab (TORCHLIGHT, Nature Medicine 2024), the first Chinese anti-PD-1 antibody to demonstrate OS benefit in this disease, and (3) a stratified analytic framework that enables quantitative comparison of treatment effect magnitude across clinical settings, ICI classes, and chemotherapy backbones—an analysis motivated by the clinically instructive divergence between IMpassion130 (nab-paclitaxel, positive) and IMpassion131 (paclitaxel, negative).

## 2 Methods

### 2.1 Protocol and registration

This systematic review and meta-analysis was conducted in accordance with the Preferred Reporting Items for Systematic Reviews and Meta-Analyses (PRISMA) 2020 statement (24). The protocol was prospectively registered with the International Prospective Register of Systematic Reviews (PROSPERO; registration number: CRD420261356428).

### 2.2 Search strategy

We systematically searched PubMed and ClinicalTrials.gov from database inception to May 2, 2026. The PubMed search strategy combined Medical Subject Headings (MeSH) terms and free-text keywords related to triple-negative breast cancer, immune checkpoint inhibitors, chemotherapy, and randomized controlled trials using Boolean operators. The complete PubMed search strategy was as follows:

((“Triple Negative Breast Neoplasms”[MeSH] OR “triple negative breast”[Title/Abstract]

OR “TNBC”[Title/Abstract])

AND

(“Immune Checkpoint Inhibitors”[MeSH] OR “Programmed Cell Death 1 Receptor”[MeSH]

OR “B7-H1 Antigen”[MeSH] OR “PD-1”[Title/Abstract] OR “PD-L1”[Title/Abstract]

OR “pembrolizumab”[Title/Abstract] OR “atezolizumab”[Title/Abstract]

OR “toripalimab”[Title/Abstract] OR “nivolumab”[Title/Abstract]

OR “durvalumab”[Title/Abstract] OR “avelumab”[Title/Abstract]

OR “immune checkpoint”[Title/Abstract] OR “immunotherapy”[Title/Abstract])

AND

(“chemotherapy”[Title/Abstract] OR “nab-paclitaxel”[Title/Abstract]

OR “paclitaxel”[Title/Abstract] OR “carboplatin”[Title/Abstract]

OR “gemcitabine”[Title/Abstract] OR “anthracycline”[Title/Abstract])

AND

(“randomized controlled trial”[Publication Type] OR “randomized”[Title/Abstract]

OR “randomised”[Title/Abstract] OR “RCT”[Title/Abstract]))

Additionally, we searched ClinicalTrials.gov from inception to May 2, 2026 using the following structured query in the Advanced Search interface: Condition/Disease = “triple negative breast cancer”; Intervention/Treatment = “pembrolizumab” OR “atezolizumab” OR “toripalimab” OR “durvalumab” OR “avelumab” OR “PD-1” OR “PD-L1”; Study Type = “Interventional (Clinical Trial)”; Phases = “Phase 2, Phase 3”; Status = “Completed, Active, not recruiting, Recruiting”. This search returned 38 records, which were exported in RIS format for screening. We also hand-searched the conference proceedings of the American Society of Clinical Oncology (ASCO), European Society for Medical Oncology (ESMO), and San Antonio Breast Cancer Symposium (SABCS) from 2020 to 2026. The reference lists of the included studies and relevant review articles were screened for additional eligible trials.

### 2.3 Inclusion and exclusion criteria

Inclusion criteria: (1) phase II or III randomized controlled trials, (2) patients with histologically confirmed triple-negative breast cancer, (3) intervention arm receiving a PD-1 or PD-L1 inhibitor combined with chemotherapy (or as monotherapy in the adjuvant setting), (4) control arm receiving chemotherapy alone, chemotherapy plus placebo, or observation, and (5) reporting at least one of the following outcomes: overall survival (OS), progression-free survival (PFS), event-free survival (EFS), invasive disease-free survival (iDFS), pathological complete response (pCR), or grade ≥3 adverse events.

Exclusion criteria: (1) single-arm trials, (2) non-randomized studies, (3) studies published only as conference abstracts without full-text availability (unless presenting a long-term follow-up of a previously fully published trial), and (4) studies reporting pCR as the sole outcome without survival data.

### 2.4 Study selection and data extraction

Two reviewers independently screened the titles and abstracts, followed by full-text assessment of potentially eligible studies. Discrepancies were resolved by consensus. For each included trial, we extracted the following data: study characteristics (trial name, phase, NCT identifier, publication year, and first author), patient demographics (sample size, age, PD-L1 status, and disease stage), treatment details (ICI agent, chemotherapy backbone, and treatment duration), and outcome data (hazard ratios with 95% confidence intervals for OS, PFS, EFS, and iDFS; odds ratios for pCR and grade ≥3 adverse events).

For survival outcomes, we preferentially extracted data from the most recent publication or conference presentation with the longest follow-up duration. When multiple PD-L1 strata were reported within the same trial, we extracted the primary analysis population as defined in the trial protocol. All HRs, ORs, and CIs were independently verified by two reviewers (BG and RZ) against the source publications.

### 2.5 Risk of bias assessment

Risk of bias was assessed using the Cochrane Risk of Bias 2 (RoB 2) tool for randomized trials (25). Five domains were evaluated: randomization process, deviations from intended interventions, missing outcome data, measurement of the outcome, and selection of the reported result. Each trial was rated as having low risk of bias, some concerns, or high risk of bias.

### 2.6 Statistical analysis

All analyses were stratified by clinical setting: metastatic (unresectable locally advanced or metastatic TNBC) and early-stage (neoadjuvant, adjuvant, or combined neoadjuvant–adjuvant strategies). Within each setting, primary analyses were conducted in the intention-to-treat (ITT) population, with prespecified subgroup analyses by PD-L1 expression status.

Hazard ratios (HRs) for time-to-event outcomes (OS, PFS, EFS, and iDFS) and odds ratios (ORs) for binary outcomes (pCR and grade ≥3 adverse events) were pooled using random-effects models with the DerSimonian–Laird estimator (26). Heterogeneity was assessed using the *I*² statistic, with values of 25%, 50%, and 75% considered indicative of low, moderate, and high heterogeneity, respectively (27), and the Cochran *Q* test (*P <*0.10 considered significant).

The prespecified subgroup analyses included (1) PD-L1 expression status (positive vs. negative, using each trial’s defined threshold), (2) ICI class (anti-PD-1 vs. anti-PD-L1), (3) chemotherapy backbone (taxane-based vs. platinum-containing vs. capecitabine), and (4) disease stage (metastatic vs. early-stage, for OS only).

Sensitivity analyses were performed by (1) sequentially excluding individual trials (leave-one-out analysis) to assess the influence of any single study, (2) comparing fixed-effect and random-effects model estimates, and (3) excluding phase II trials to determine the robustness of findings restricted to phase III evidence.

Funnel plots were used for visual inspection of publication bias. Formal Egger’s testing was not performed because no outcome had 10 or more contributing trials. All statistical analyses were performed using R (version 4.3.0) with the meta and metafor packages. Two-sided *P*-values <0.05 were considered statistically significant.

## 3 Results

### 3.1 Study selection

The systematic literature search identified 197 records in PubMed, 38 records in ClinicalTrials.gov, and 24 additional records through hand-searching of conference proceedings and reference lists. After removing duplicates (*n* = 19), 240 unique records underwent title and abstract screening, of which 193 were excluded. A total of 47 full-text articles were assessed for eligibility, whereas 37 were excluded for the following reasons: single-arm design (*n* = 11), conference abstract without full-text publication (*n* = 8), subgroup or secondary analysis of included trials (*n* = 7), non-TNBC population (*n* = 5), review or commentary (*n* = 4), and pCR reported as the sole outcome without survival data (*n* = 2). A total of 10 RCTs met all of the inclusion criteria (**Figure 1**): five in the metastatic setting (KEYNOTE-355, IMpassion130, IMpassion131, IMpassion132, and TORCHLIGHT) and five in the early-stage setting (KEYNOTE-522, IMpassion031, GeparNuevo, NeoTRIP, and A-BRAVE).

**Figure 1:**
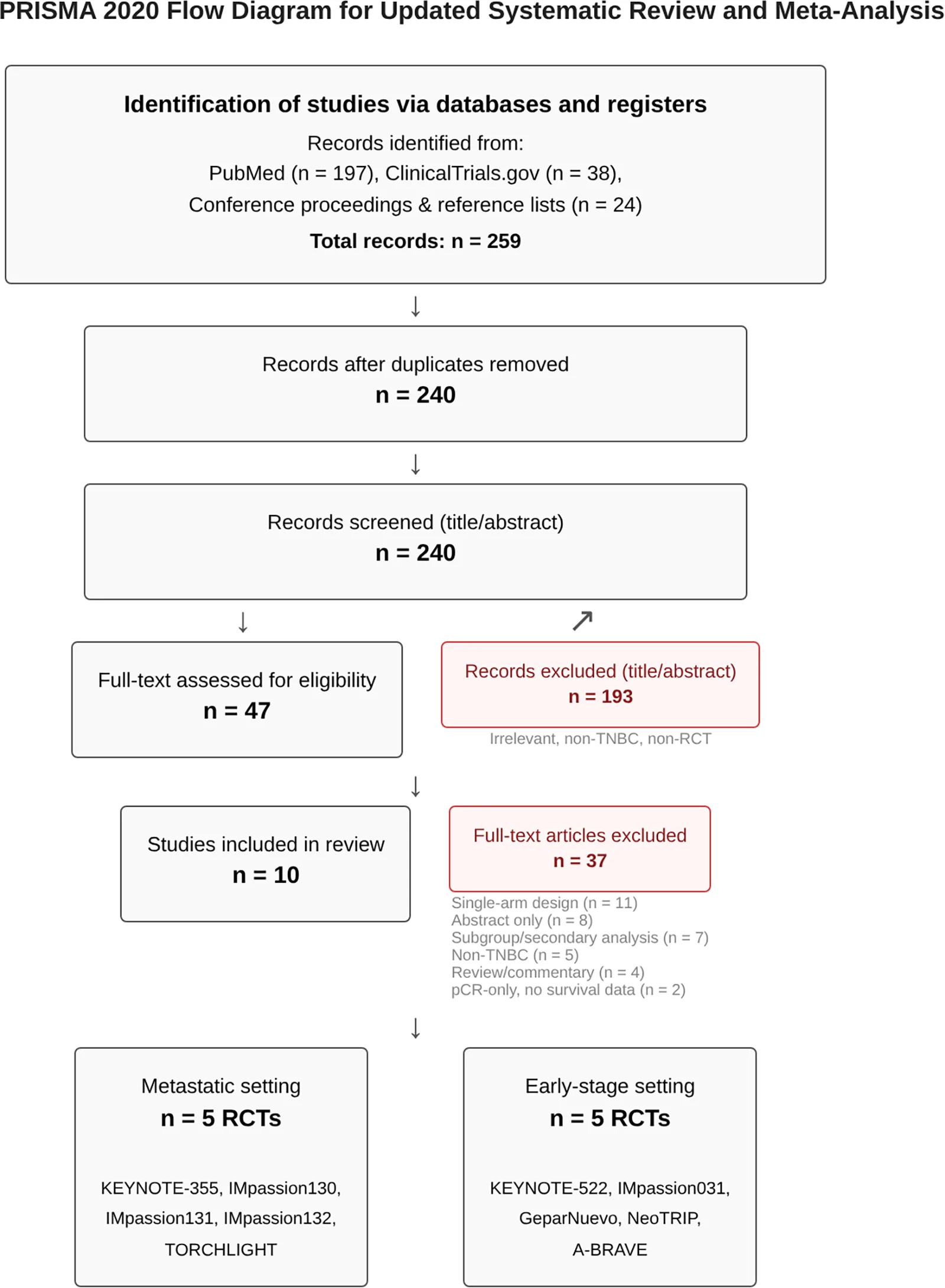
PRISMA 2020 flow diagram. The systematic search of PubMed (*n =* 197) and ClinicalTrials.gov (*n* = 38) and hand-searching of conference proceedings and reference lists (*n* = 24) identified 259 records. After removal of duplicates (*n* = 19), 240 unique records underwent title and abstract screening, of which 193 were excluded. A total of 47 full-text articles were assessed for eligibility; 37 were excluded for single-arm design (*n* = 11), conference abstract without full-text publication (*n* = 8), subgroup or secondary analysis of an included trial (*n* = 7), non-TNBC population (*n* = 5), review or commentary (*n* = 4), and pCR reported as the sole outcome without survival data (*n* = 2). A total of 10 RCTs met all inclusion criteria and were included in the systematic review and meta-analysis (metastatic setting: *n* = 5; early-stage setting: *n* = 5).

### 3.2 Study characteristics

The 10 included RCTs randomized a total of 5,953 patients (**Table 1**; **Supplementary Table S2** provides detailed baseline characteristics). In the metastatic setting (*n* = 3,526), pembrolizumab was evaluated in KEYNOTE-355 (*n* = 847), atezolizumab in IMpassion130 (*n* = 902), IMpassion131 (*n* = 651), and IMpassion132 (*n* = 595), and toripalimab in TORCHLIGHT (*n* = 531). Chemotherapy backbones comprised nab-paclitaxel (IMpassion130 and TORCHLIGHT), paclitaxel (IMpassion131), and gemcitabine plus carboplatin or capecitabine (IMpassion132); KEYNOTE-355 used investigator-choice chemotherapy and was excluded from backbone subgroup analyses. In the early-stage setting (*n* = 2,427), pembrolizumab was evaluated in KEYNOTE-522 (*n* = 1,174), atezolizumab in IMpassion031 (*n* = 333) and NeoTRIP (*n* = 280), durvalumab in GeparNuevo (*n* = 174), and avelumab in A-BRAVE (*n* = 466).

**Table 1:** Baseline characteristics of the included randomized controlled trials.

Trial	Phase	NCT	Setting	Patients (*N*)	Experimental arm	Control arm	Primary endpoint(s)	PD-L1 assay	Key efficacy (latest follow-up)
Metastatic setting									
KEYNOTE-355	III	NCT02819518	1L mTNBC	847	Pembro 200 mg Q3W + chemo (nab-PTX/PTX/Gem+Cb)	Placebo + chemo	PFS and OS (CPS ≥ 10, CPS ≥ 1, ITT)	22C3	CPS ≥ 10: OS HR 0.73 (0.55–0.95); PFS HR 0.65 (0.49–0.86)
IMpassion130	III	NCT02425891	1L mTNBC	902	Atezo 840 mg Q2W + nab-PTX 100 mg/m²	Placebo + nab-PTX	PFS and OS (ITT, PD-L1 IC+)	SP142	ITT: OS HR 0.87 (0.75–1.02); PD-L1 IC+: OS HR 0.67 (0.53–0.86)
IMpassion131	III	NCT03125902	1L mTNBC	651	Atezo 840 mg Q2W + PTX 90 mg/m²	Placebo + PTX	PFS (PD-L1+)	SP142	PD-L1+: PFS HR 0.82 (0.60–1.12); OS HR 1.11 (negative)
IMpassion132	III	NCT03371017	Early-relapse mTNBC	595	Atezo 1,200 mg Q3W + chemo (Gem+Cb/Cape)	Placebo + chemo	OS (PD-L1+)	SP142	PD-L1+: OS HR 0.93 (0.73–1.20); PFS HR ~1.0
TORCHLIGHT	III	NCT04085276	1L mTNBC	531	Toripalimab 240 mg Q3W + nab-PTX 125 mg/m²	Placebo + nab-PTX	PFS and OS (PD-L1+, ITT)	JS311	ITT: OS HR 0.69 (0.51–0.93); PD-L1+ OS HR 0.62 (0.41–0.91)
Early-stage setting									
KEYNOTE-522	III	NCT03036488	Stage II–III TNBC	1,174	Pembro + Cb/PTX → Pembro + AC → adj Pembro	Placebo + Cb/PTX → Placebo + AC → adj Placebo	pCR, EFS	22C3	pCR: 64.8% vs. 51.2%; EFS HR 0.65 (0.51–0.83); OS HR 0.66 (0.50–0.87)
IMpassion031	III	NCT03197935	Stage II–III TNBC	333	Atezo + nab-PTX → Atezo + AC → adj Atezo	Placebo + nab-PTX → placebo + AC	pCR	SP142	pCR: 57.6% vs. 41.1%; OR 1.95 (1.33–2.86)
GeparNuevo	II	NCT02685059	Early TNBC	174	Durva 1.5 g Q4W + nab-PTX → EC	Placebo + nab-PTX → EC	pCR	SP263	pCR: 53.4% vs. 44.2%; 7-year iDFS HR 0.56 (0.32–0.99); OS HR 0.33 (0.14–0.79)
NeoTRIP	III	NCT02620280	Early high-risk TNBC	280	Atezo + Cb/nab-PTX	Cb/nab-PTX	EFS	SP142	pCR: 48.6% vs. 44.4%; OR 1.18 (0.74–1.90); EFS HR 1.08 (0.76–1.54) (28)
A-BRAVE	III	NCT02926196	Adjuvant high-risk TNBC	466	Avelumab 10 mg/kg Q2W × 1 year	Observation	DFS (ITT)	73–10	DFS HR 0.81 (0.61–1.09); OS HR 0.66 (0.45–0.97)

1L, first-line; mTNBC, metastatic triple-negative breast cancer; Pembro, pembrolizumab; Atezo, atezolizumab; Durva, durvalumab; nab-PTX, nab-paclitaxel; PTX, paclitaxel; Gem, gemcitabine; Cb, carboplatin; AC, doxorubicin/cyclophosphamide; EC, epirubicin/cyclophosphamide; Cape, capecitabine; adj, adjuvant; CPS, combined positive score; IC, immune cell; pCR, pathological complete response; EFS, event-free survival; iDFS, invasive disease-free survival; DFS, disease-free survival; OS, overall survival; PFS, progression-free survival; HR, hazard ratio; OR = odds ratio.

### 3.3 Risk of bias

Eight trials were rated as having low risk of bias (**Supplementary Table S1**). NeoTRIP and A-BRAVE—the two open-label designs—were rated as having some concerns in domain 2 (deviations from intended interventions, owing to lack of blinding) and domain 4 (outcome measurement). No trial was rated as high risk of bias. KEYNOTE-355, IMpassion130, IMpassion131, IMpassion132, KEYNOTE-522, TORCHLIGHT, IMpassion031, and GeparNuevo were double-blind and placebo-controlled.

### 3.4 Meta-analysis: metastatic setting

#### 3.4.1 Overall survival

Five trials reported OS data in the metastatic setting (*n* = 3,526). In the ITT population, ICI-chemotherapy improved OS (pooled HR 0.85, 95% CI 0.74–0.96, *P* = 0.013; *I*² = 27%, *P* heterogeneity = 0.24; **Figure 2A**). Individual trial OS data were extracted from the KEYNOTE-355 final analysis (22), the IMpassion130 final OS analysis (29), IMpassion131 (8), IMpassion132 (30), and TORCHLIGHT (23). Under the Hartung–Knapp–Sidik–Jonkman (HKSJ) adjustment, the CI widened (0.70–1.03, *P* = 0.076) and the 95% prediction interval was 0.63–1.13, reflecting between-study dispersion.

**Figure 2:**
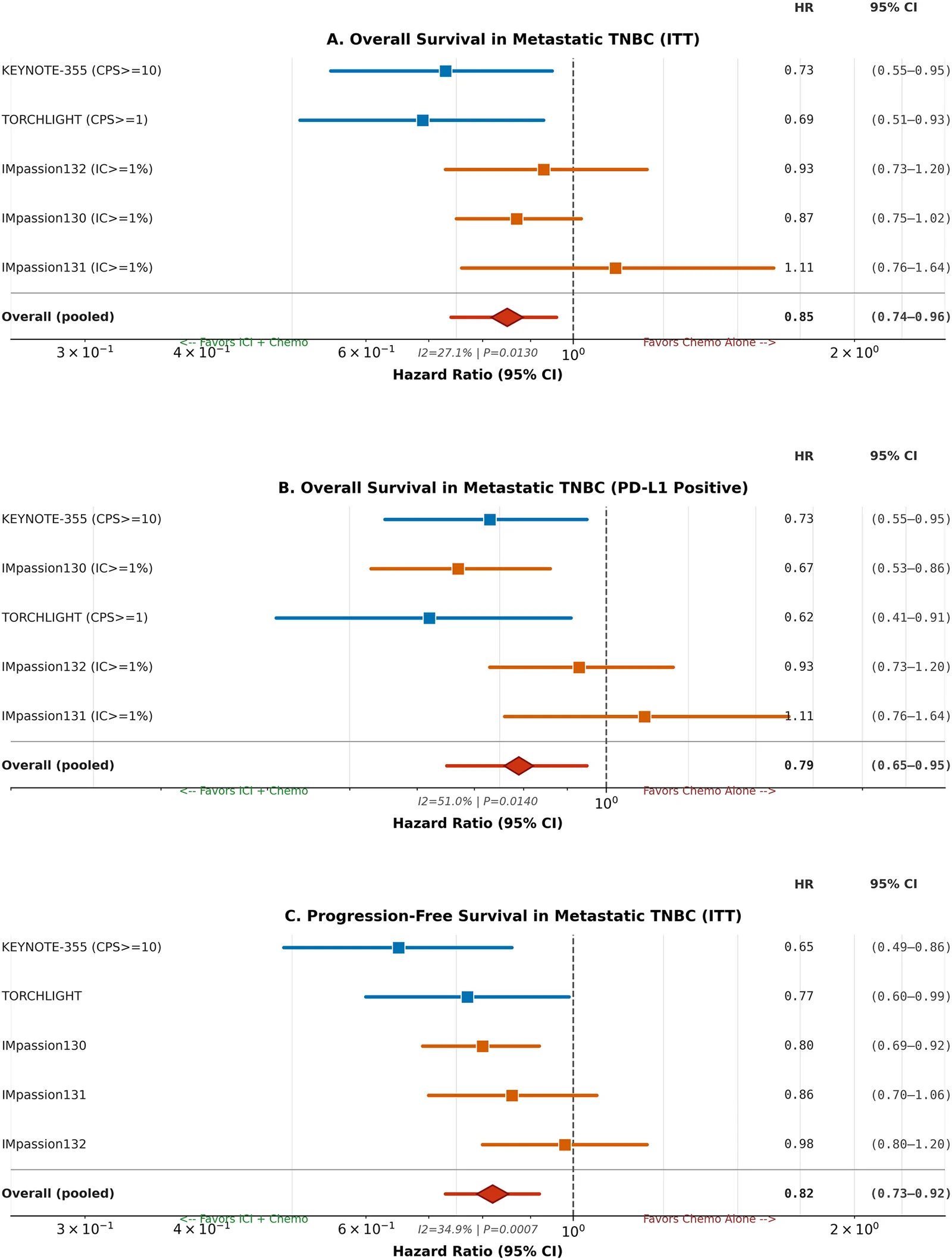
Forest plots for efficacy outcomes in the metastatic setting. **(A)** Overall survival in the primary OS analysis population across five RCTs (*n* = 3,526). Pooled HR 0.85 (95% CI 0.74–0.96; *P* = 0.013; *I*² = 27%) using a random-effects (DerSimonian-Laird) model; the HKSJ-adjusted CI was 0.70–1.03. **(B)** Overall survival in the PD-L1-positive subgroup (defined per trial’s primary PD-L1 cutoff). Pooled HR 0.79 (95% CI 0.65–0.95; *P* = 0.014; *I*² = 51%). **(C)** Progression-free survival across five RCTs. Pooled HR 0.82 (95% CI 0.73–0.92; *P* = 0.0007; *I*² = 35%). Blue squares denote anti-PD-1 agents, and orange squares denote anti-PD-L1 agents. Square size is proportional to study weight. Horizontal lines represent 95% CIs. The red diamond represents the pooled random-effects estimate. The dashed vertical line at HR = 1.0 indicates the line of no effect.

In the PD-L1-positive population, the OS benefit remained significant under the DL model (pooled HR 0.79, 95% CI 0.65–0.95, *P* = 0.014; *I*² = 51%, *P* heterogeneity = 0.086; **Figure 2B**), but the HKSJ-adjusted CI crossed 1.0 (0.60–1.04, *P* = 0.077), indicating sensitivity to the choice of variance estimator with five studies. The largest effect sizes were observed in TORCHLIGHT (PD-L1 CPS ≥ 1: HR 0.62, 95% CI 0.41–0.91), IMpassion130 (PD-L1 IC ≥ 1%: HR 0.67, 95% CI 0.53–0.86), and KEYNOTE-355 (CPS ≥ 10: HR 0.73, 95% CI 0.55–0.95).

Conversely, in the PD-L1-negative population, no significant OS benefit was observed (pooled HR 0.96, 95% CI 0.81–1.13, *P* = 0.61; *I*² = 0%).

#### 3.4.2 Progression-free survival

PFS was significantly improved under both DL and HKSJ models (pooled HR 0.82, 95% CI 0.73–0.92, *P* = 0.0007; HKSJ 0.69–0.97, *P* = 0.031; *I*² = 35%; **Figure 2C**). The PFS benefit was greater in PD-L1-positive populations (pooled HR 0.73, 95% CI 0.63–0.84) compared with PD-L1-negative populations (pooled HR 0.88, 95% CI 0.77–1.01).

Notably, IMpassion131—the only trial using solvent-based paclitaxel rather than nab-paclitaxel—showed no PFS benefit (ITT: HR 0.86, 95% CI 0.70–1.06), contrasting with the significant benefits observed across trials employing nab-paclitaxel backbones.

#### 3.4.3 Subgroup analyses in the metastatic setting

ICI class (exploratory): Anti-PD-1 agents (pembrolizumab and toripalimab) showed a numerically greater OS benefit (pooled HR 0.71, 95% CI 0.58–0.87) than anti-PD-L1 agents (atezolizumab; pooled HR 0.91, 95% CI 0.80–1.03; nominal between-subgroup *P* = 0.045). However, this difference was not robust: leave-one-out sensitivity analyses showed that the subgroup *P*-value became non-significant after the exclusion of any single trial (*P* = 0.074–0.156), and the backbone-matched comparison (TORCHLIGHT vs. IMpassion130, both nab-paclitaxel-based) showed no significant difference (*P* = 0.178). These findings indicate that the apparent anti-PD-1 versus anti-PD-L1 difference is trial-driven rather than mechanism-driven, and no mechanistic superiority of PD-1 over PD-L1 blockade should be inferred. A definitive comparison would require a head-to-head randomized trial.

Chemotherapy backbone: To avoid unit-of-analysis problems from KEYNOTE-355’s investigator-choice design, the backbone analysis was restricted to trials with uniform chemotherapy assignment. The nab-paclitaxel-based trials (IMpassion130 and TORCHLIGHT) yielded a pooled OS HR of 0.72 (95% CI 0.58–0.89), the paclitaxel-based trial (IMpassion131) showed no benefit (HR 1.11, 0.76–1.64), and the gemcitabine/carboplatin or capecitabine trial (IMpassion132) showed no benefit (HR 0.93, 0.73–1.20). The test for subgroup differences was *Q* = 5.21, df = 2, *P* = 0.074. This comparison is ecological and non-randomized.

#### 3.4.4 Sensitivity analyses in the metastatic setting

Stratified leave-one-out analysis confirmed that no single trial drove the pooled estimate (**Supplementary Figure S2**). In the metastatic setting, HRs ranged from 0.82 to 0.88; the estimate was most sensitive to the exclusion of TORCHLIGHT (HR 0.88). In the early-stage setting, HRs ranged from 0.61 to 0.67; the exclusion of GeparNuevo (Phase II, HR 0.33) attenuated the pooled HR to 0.67 (95% CI 0.54–0.82, *P* < 0.001), but the benefit remained significant. The exclusion of KEYNOTE-522 broadened the CI substantially (HR 0.61, 0.45–0.84), reflecting its dominant weight. Fixed-effect and random-effects estimates were consistent across all primary outcomes (**Supplementary Table S3**).

### 3.5 Meta-analysis: early-stage setting

#### 3.5.1 Event-free survival/invasive disease-free survival

Five trials reported EFS or iDFS data (*n* = 2,427). ICIs significantly improved EFS/iDFS (pooled HR 0.75, 95% CI 0.63–0.90, *P* = 0.0015; *I*² = 0.2%; HKSJ 0.58–0.97, *P* = 0.036; **Figure 3A**).

**Figure 3:**
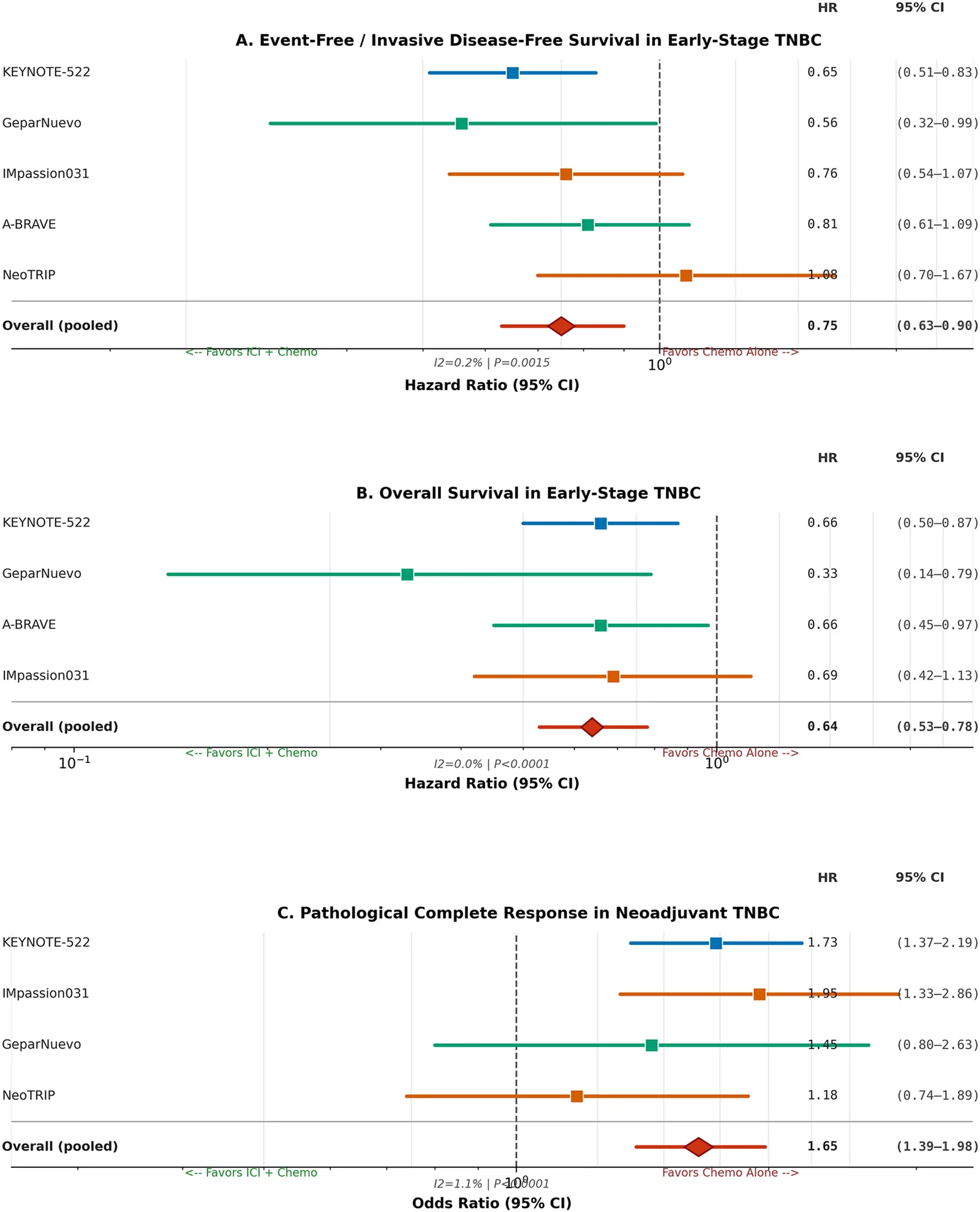
Forest plots for efficacy outcomes in the early-stage setting. **(A)** Event-free survival, invasive disease-free survival, or disease-free survival across five RCTs (*n* = 2,427). Pooled HR 0.75 (95% CI 0.63–0.90; *P* = 0.0015; *I*² = 0.2%). **(B)** Overall survival across four RCTs. Pooled HR 0.64 (95% CI 0.53–0.78; *P* < 0.0001; *I*² = 0.0%). **(C)** Pathological complete response (ypT0/Tis ypN0) across four neoadjuvant RCTs (*n* = 1,961). Pooled odds ratio 1.65 (95% CI 1.39–1.98; *P* < 0.0001; *I*² = 1.1%). Blue, anti-PD-1; orange, anti-PD-L1; green, durvalumab/avelumab. The abbreviations are as in **Figure 2**.

KEYNOTE-522 demonstrated the most robust EFS benefit (HR 0.65, 95% CI 0.51–0.83) (11), followed by GeparNuevo (7-year iDFS HR 0.56, 95% CI 0.32–0.99) (31). A-BRAVE showed a numerical but non-significant DFS improvement (HR 0.81, 95% CI 0.61–1.09) (15). NeoTRIP did not demonstrate an EFS benefit (HR 1.08) (28).

#### 3.5.2 Overall survival

Four trials reported OS data. ICIs significantly improved OS (pooled HR 0.64, 95% CI 0.53–0.78, *P* < 0.0001; *I*² = 0%; HKSJ 0.48–0.86, *P* = 0.016; **Figure 3B**).

KEYNOTE-522, with the longest follow-up (median 75.1 months), demonstrated an OS HR of 0.66 (95% CI 0.50–0.87), corresponding to a 5-year OS rate of 86.6% vs. 81.7% (11). GeparNuevo, with 7-year follow-up, showed a large OS benefit (HR 0.33, 95% CI 0.14–0.79) (31), with an absolute OS improvement of 16.9% at 7 years. A-BRAVE demonstrated a significant OS benefit in the adjuvant setting (HR 0.66, 95% CI 0.45–0.97) (15).

#### 3.5.3 Pathological complete response

Four trials reported pCR data (*n* = 1,961). ICIs significantly increased pCR (pooled OR 1.65, 95% CI 1.39–1.98, *P* < 0.0001; *I*² = 1%; HKSJ 1.24–2.21, *P* = 0.012; **Figure 3C**).

KEYNOTE-522 demonstrated the most pronounced pCR improvement (64.8% vs. 51.2%, OR 1.73). IMpassion031 showed a similar magnitude of benefit (57.6% vs. 41.1%, OR 1.95). NeoTRIP did not demonstrate a significant pCR improvement (48.6% vs. 44.4%, OR 1.18). A prespecified sensitivity analysis stratified by platinum-containing (KEYNOTE-522 and NeoTRIP) versus non-platinum-containing (IMpassion031 and GeparNuevo) neoadjuvant backbones showed comparable benefit (pooled OR 1.51 [1.06–2.16] vs. 1.79 [1.30–2.47], *P* interaction = 0.494), indicating that the pCR benefit is attributable to ICI addition rather than to the chemotherapy backbone alone.

#### 3.5.4 PD-L1 subgroup in early stage

Treatment strategy: A prespecified subgroup analysis by treatment strategy was not significant (*P* = 0.619), supporting pooling: neoadjuvant-plus-adjuvant ICI (KEYNOTE-522 and IMpassion031) HR 0.69 (0.56–0.84), neoadjuvant-only ICI (GeparNuevo and NeoTRIP) HR 0.80 (0.42–1.52), and adjuvant-only ICI (A-BRAVE) HR 0.81 (0.61–1.08). In KEYNOTE-522, the OS benefit was consistent across PD-L1 subgroups (CPS ≥ 1: HR 0.70; CPS < 1: HR 0.51), suggesting that PD-L1 may be less predictive in treatment-naive early-stage disease than in metastatic disease.

#### 3.5.5 Sensitivity analyses in early stage

Exclusion of the phase II GeparNuevo trial from the early-stage OS meta-analysis yielded a pooled HR of 0.67 (95% CI 0.54–0.82, *P* < 0.001), confirming that the overall OS benefit is not solely attributable to this small trial. For EFS/iDFS, exclusion of GeparNuevo yielded HR 0.77 (0.64–0.93, *P* = 0.007). Exclusion of KEYNOTE-522 from the OS analysis yielded HR 0.61 (0.45–0.84), reflecting this trial’s dominant weight.

### 3.6 Safety

Immune-related adverse events (irAEs) of any grade were significantly more frequent with ICI-containing regimens in both the metastatic (pooled OR 2.45, 95% CI 1.85–3.25) and early-stage settings (pooled OR 2.73, 95% CI 1.91–3.90). Grade ≥3 treatment-related adverse events were not significantly increased in the metastatic setting (pooled OR 1.08, 95% CI 0.93–1.25) and only modestly increased in the early-stage setting (pooled OR 1.18, 95% CI 1.02–1.37). Treatment-related deaths were rare and not significantly different between arms. These safety findings are consistent with the patient-reported quality-of-life results from KEYNOTE-522, which showed no clinically meaningful deterioration with the addition of pembrolizumab to chemotherapy (32).

## 4 Discussion

This updated systematic review and meta-analysis, incorporating 10 RCTs and 5,953 patients with the longest available follow-up, provides several clinically important findings.

First, ICIs combined with chemotherapy provide consistent and clinically meaningful OS benefit in both metastatic and early-stage TNBC. In the metastatic setting, the pooled OS HR of 0.85 (95% CI 0.74–0.96; *I*² = 27%) corroborates prior meta-analyses. Under the HKSJ adjustment, which is recommended with few-study meta-analyses, the CI widened (0.70–1.03, *P* = 0.076), and the 95% prediction interval (0.63–1.13) indicates that the true effect in a future study could range from clinically meaningful benefit to minimal effect. This method sensitivity should be interpreted in light of the consistent direction of effect across all five trials. Our analysis incorporates TORCHLIGHT, demonstrating that toripalimab confers OS and PFS benefits comparable to pembrolizumab. In the early-stage setting, the pooled OS HR of 0.64 (0.53–0.78; *I*² = 0%) remained significant under HKSJ (0.48–0.86, *P* = 0.016), driven by mature KEYNOTE-522 data and the provocative GeparNuevo 7-year results.

Second, PD-L1 expression remains the most clinically useful, albeit imperfect, biomarker for patient selection in the metastatic setting. In PD-L1-positive patients, the OS HR of 0.79 represents a 21% reduction in the risk of death. In PD-L1-negative patients, the absence of significant OS benefit (HR 0.96) underscores the critical unmet need for more effective therapies. However, the predictive value of PD-L1 appears attenuated in the early-stage setting, where KEYNOTE-522 demonstrated OS benefit even in PD-L1-negative patients (CPS < 1: HR 0.51). This discordance may reflect fundamental differences in tumor immunobiology between the primary tumor microenvironment and the immunosuppressive metastatic niche (33).

Third, the choice of chemotherapy partner critically modulates ICI efficacy (34). The IMpassion130-IMpassion131 discordance—wherein atezolizumab combined with nab-paclitaxel improved OS (HR 0.67), but the same anti-PD-L1 antibody combined with solvent-based paclitaxel showed an OS trend favoring the control arm (HR 1.11)—represents the most striking example. Miles et al. (the IMpassion131 investigators) explicitly noted in their primary publication that the mandatory dexamethasone premedication with solvent-based paclitaxel—absent with nab-paclitaxel—constituted a key design difference warranting investigation (8). This corticosteroid hypothesis is mechanistically supported by Giles et al., who demonstrated that dexamethasone attenuates CD28 co-stimulatory signaling in T cells, an effect partially reversible by CTLA-4 but not PD-1 blockade (35). Complementary mechanisms include enhanced tumor antigen presentation through albumin-mediated delivery of nab-paclitaxel to tumor-associated macrophages (36) and immunomodulatory effects of Cremophor EL, the solvent vehicle in conventional paclitaxel (37). In our analysis, nab-paclitaxel-based trials (IMpassion130 and TORCHLIGHT) yielded a pooled OS HR of 0.72 (95% CI 0.58–0.89), whereas trials predominantly employing non-nab-paclitaxel backbones yielded a pooled OS HR of 0.91 (95% CI 0.79–1.04). While this between-trial comparison cannot establish causality, the consistency of the pattern—combined with the mechanistic rationale—supports the recommendation that future phase III trials of novel ICI combinations in TNBC should prospectively consider the immunomodulatory properties of the chosen chemotherapy partner and the potential confounding effect of corticosteroid premedication.

Chemotherapy resistance represents a further mechanistic layer relevant to chemo-immunotherapy efficacy. TNBC chemoresistance arises through cancer stem cell induction, ATP-binding cassette (ABC) transporter-mediated drug efflux, hypoxia, and apoptosis evasion (38) and is further modulated by non-coding RNA circuitry such as circPLK1-mediated anthracycline resistance (39). These mechanisms may attenuate ICI benefit through several pathways: hypoxia promotes an immunosuppressive tumor microenvironment that blunts T-cell function; ABC-transporter-mediated efflux can reduce chemotherapy-induced immunogenic cell death, thereby diminishing the release of tumor antigens that potentiate ICI response; and cancer stem cell induction may select for a less immunogenic tumor phenotype. These predictions are consistent with the negative outcomes observed in the early-relapse population of IMpassion132 (relapse within 12 months of curative-intent chemotherapy, a proxy for chemoresistant disease) and with the attenuated benefit observed in heavily pretreated populations. This framework suggests that chemoresistance status may be an underappreciated modifier of chemo-immunotherapy efficacy, warranting prospective stratification in future trials.

Fourth, the long-term survival data reveal durable benefit. KEYNOTE-522’s 5-year OS rate of 86.6% with pembrolizumab vs. 81.7% without represents a paradigm shift. The GeparNuevo 7-year data—demonstrating 16.9% absolute OS improvement despite neoadjuvant-only durvalumab without adjuvant continuation—raise the provocative question of whether adjuvant-phase immunotherapy is always necessary.

Fifth, the exploratory subgroup comparison between anti-PD-1 and anti-PD-L1 agents (nominal *P* = 0.045) did not survive leave-one-out sensitivity analysis: exclusion of any single trial rendered the subgroup difference non-significant (*P* = 0.074-0.156), and the backbone-matched comparison showed no significant difference (*P* = 0.178). The pharmacologic distinction—PD-1 inhibitors block both PD-L1 and PD-L2, whereas PD-L1 inhibitors spare PD-L2/PD-1 signaling (33)—remains mechanistically plausible but is not supported by this trial-level analysis. The apparent difference is trial-driven rather than mechanism-driven, and no superiority of PD-1 over PD-L1 blockade should be inferred from these data.

Sixth, the safety data are reassuring. While immune-related AEs were more frequent with ICIs (any-grade OR 2.45–2.73), the absolute increase in grade ≥3 treatment-related AEs was modest (OR 1.08–1.18). Treatment-related deaths were rare and comparable between arms.

### 4.1 Limitations

This analysis has several limitations. First, our search was restricted to PubMed, ClinicalTrials.gov, conference proceedings, and reference lists; an Embase search was subsequently performed and yielded no additional eligible trials, but the absence of CENTRAL searching is noted. Second, the number of included trials is moderate (*n* = 10), and with four to five trials per analysis, the DL estimator may underestimate between-study variance—motivating our addition of HKSJ adjustment and prediction intervals. Third, cross-trial heterogeneity in PD-L1 assay platforms (SP142, 22C3, JS311, 73-10, and SP263), positivity thresholds (IC ≥ 1%, CPS ≥ 1, and CPS ≥ 10), chemotherapy backbones, and patient populations limits precision and generalizability. The PD-L1-positive pooled analysis mixes non-equivalent assays: KEYNOTE-355’s CPS ≥10 population is not interchangeable with the CPS ≥1 or IC ≥1% populations of other trials. Fourth, the striking OS benefit in GeparNuevo (HR 0.33) warrants caution given its phase II design and small sample size (*n* = 174). Fifth, the early-stage EFS/iDFS analysis pools heterogeneous endpoints (EFS, iDFS, and DFS) and treatment strategies (neoadjuvant + adjuvant, neoadjuvant-only, adjuvant-only), though the strategy subgroup analysis was non-significant (*P* = 0.619). Sixth, the early-stage OS analysis is dominated by KEYNOTE-522. Seventh, not all trials have reported final OS analyses. Eighth, the A-BRAVE trial evaluated adjuvant avelumab monotherapy versus observation rather than chemotherapy combined with an ICI; its inclusion is acknowledged in the revised title. Finally, AI-assisted data extraction was independently verified by two human reviewers (BG and RZ) against all source publications.

### 4.2 Implications for clinical practice and research

These findings support current guideline recommendations for pembrolizumab-based regimens in both early-stage (KEYNOTE-522) and PD-L1-positive metastatic TNBC (KEYNOTE-355). The TORCHLIGHT data suggest toripalimab as an additional option. In absolute terms, the addition of pembrolizumab improved 5-year OS by 4.9 percentage points (86.6% vs. 81.7%) in early-stage TNBC and added a median OS gain of 6.9 months in metastatic PD-L1-positive disease. The pCR absolute gain was 13.6 percentage points. The GeparNuevo 7-year absolute OS gain was 16.9 percentage points, though the phase II design limits generalizability. The absolute increase in grade ≥3 treatment-related AEs was modest (pooled OR 1.08–1.18), suggesting a favorable benefit–toxicity ratio. A backbone-stratified pCR sensitivity analysis showed comparable benefit regardless of platinum use (*P* interaction = 0.494).

The consistently negative outcomes in PD-L1-negative metastatic TNBC highlight a critical unmet need. Novel strategies—including antibody–drug conjugates (sacituzumab govitecan), bispecific antibodies (BNT327/PM8002), and PARP inhibitor combinations in BRCA-mutated populations—warrant prioritized investigation.

The observation that ICI benefit extends beyond pCR attainment in KEYNOTE-522 suggests that adjuvant-phase immunotherapy plays a critical role in eliminating micrometastatic disease—contrasting with GeparNuevo, where neoadjuvant-only durvalumab achieved substantial OS benefit without adjuvant continuation.

## 5 Conclusions

This updated systematic review and meta-analysis, incorporating 5,953 patients from 10 RCTs with the longest available follow-up, confirms that PD-1/PD-L1 inhibitors confer clinically meaningful survival benefit in both early-stage and metastatic TNBC. The magnitude of benefit is greatest in PD-L1-positive populations in the metastatic setting, while PD-L1 status appears less determinative in the treatment-naive early-stage context. The chemotherapy backbone critically modulates immunotherapeutic efficacy. Although the HKSJ-adjusted CIs for metastatic OS cross 1.0, reflecting the limited number of trials and the influence of two negative atezolizumab studies, the consistent direction of effect, the significant benefit under HKSJ in the early-stage setting, and the robust pCR improvement provide convergent evidence supporting ICI use. As the field moves toward next-generation strategies, the evidence synthesized here provides a benchmark against which emerging therapies should be measured.

## Data Availability

The original contributions presented in the study are included in the article/**Supplementary Material**. Further inquiries can be directed to the corresponding author.

## Supplementary material

Supplementary Table 1 Risk of bias assessment (Cochrane RoB 2). All eight double-blind, placebo-controlled trials were rated as low risk of bias across all five domains. NeoTRIP and A-BRAVE (open-label) were rated as having “some concerns” in Domain 2 and Domain 4. No trial was rated as high risk of bias.

Supplementary Table 2 Detailed trial characteristics. Comprehensive baseline characteristics including median age, ECOG performance status, PD-L1 positivity rates, prior treatment history, and detailed chemotherapy regimens across all ten included trials.

Supplementary Table 3 Fixed-effect vs. random-effects model comparison.

Supplementary Table 4 GRADE Certainty of evidence summary.

Supplementary Table 5 Trial-level data extraction dataset.

Supplementary Table 6 Population definitions for pooled analyses.

Supplementary Table 7 Leave-one-out sensitivity analysis of anti-PD-1 vs. Anti-PD-L1 subgroup comparison.

Supplementary Figure 1 Funnel plots. Funnel Plots for Assessment of Publication Bias **(A)** Metastatic OS (5 trials); **(B)** Metastatic PFS (5 trials); **(C)** Early-stage EFS/iDFS (5 trials); **(D)** pCR (OR, 4 trials). Pseudo-95% confidence limits are shown as dashed lines with the gray shaded region representing the 95% confidence region. Each point represents one trial. Given fewer than ten trials per analysis, formal Egger’s testing was not performed per our prespecified methodological criterion; visual inspection reveals no gross asymmetry suggestive of small-study effects.

Supplementary Figure 2 Leave-one-out sensitivity analysis. Leave-One-Out Sensitivity Analysis Pooled HR and 95% CI for overall survival recalculated after sequential exclusion of each individual trial, stratified by setting (metastatic OS: 5 trials; early-stage OS: 4 trials). The red diamond represents the primary pooled estimate with all trials included. The pooled HR remained statistically significant across all exclusion scenarios, confirming the robustness of the primary OS analysis.
