# Supplementary material for: PD-1/PD-L1 inhibitors combined with chemotherapy or as monotherapy versus chemotherapy alone or observation in triple-negative breast cancer: a systematic review and updated meta-analysis incorporating long-term survival data
Source: Front Immunol. 2026 Oct 6;17:1958007. doi: 10.3389/fimmu.2026.1958007 (PMC13680397; doi:10.3389/fimmu.2026.1958007)
Supplement: Supplementary file 1 [file DataSheet1.docx]

# Supplementary Materials

**Manuscript:** PD-1/PD-L1 Inhibitors Combined with Chemotherapy or as Monotherapy versus Chemotherapy Alone or Observation in Triple-Negative Breast Cancer: A Systematic Review and Updated Meta-Analysis Incorporating Long-Term Survival Data

## Supplementary Table 1. Risk of Bias Assessment (Cochrane RoB 2)

| Trial | D1 Randomization | D2 Deviations | D3 Missing Data | D4 Measurement | D5 Selective Reporting | Overall |
| --- | --- | --- | --- | --- | --- | --- |
| **Metastatic** |  |  |  |  |  |  |
| KEYNOTE-355 | Low | Low | Low | Low | Low | Low |
| IMpassion130 | Low | Low | Low | Low | Low | Low |
| IMpassion131 | Low | Low | Low | Low | Low | Low |
| IMpassion132 | Low | Low | Low | Low | Low | Low |
| TORCHLIGHT | Low | Low | Low | Low | Low | Low |
| **Early-Stage** |  |  |  |  |  |  |
| KEYNOTE-522 | Low | Low | Low | Low | Low | Low |
| IMpassion031 | Low | Low | Low | Low | Low | Low |
| GeparNuevo | Low | Low | Low | Low | Low | Low |
| NeoTRIP | Low | Some concerns | Low | Some concerns | Low | Some concerns |
| A-BRAVE | Low | Some concerns | Low | Some concerns | Low | Some concerns |

**Notes:** D2 (deviations from intended interventions): NeoTRIP and A-BRAVE were open-label designs; lack of blinding may have influenced clinical management. D4 (outcome measurement): pCR assessment (NeoTRIP) and DFS ascertainment (A-BRAVE) could theoretically be influenced by knowledge of treatment assignment. All trials used central/stratified randomization (D1), had <10% loss to follow-up (D3), and reported prespecified primary endpoints consistently with protocols registered on ClinicalTrials.gov (D5).

## Supplementary Table 2. Detailed Baseline Characteristics of Included Trials

### Metastatic Setting

| Characteristic | KEYNOTE-355 | IMpassion130 | IMpassion131 | IMpassion132 | TORCHLIGHT |
| --- | --- | --- | --- | --- | --- |
| Phase | III | III | III | III | III |
| Blinding | Double-blind | Double-blind | Double-blind | Double-blind | Double-blind |
| NCT | NCT02819518 | NCT02425891 | NCT03125902 | NCT03371017 | NCT04085276 |
| N | 847 | 902 | 651 | 595 | 531 |
| Countries | 29 | 41 | 28 | 28 | China |
| Median Age (yr) | 53 | 55 | 54 | 53 | 51 |
| ECOG 0 (%) | 58 | 58 | 57 | 52 | 36 |
| Visceral mets (%) | ~80 | ~78 | ~77 | ~75 | ~72 |
| De novo metastatic (%) | ~30 | ~33 | ~35 | 0 | ~38 |
| PD-L1 assay | 22C3 (CPS) | SP142 (IC) | SP142 (IC) | SP142 (IC) | JS311 (CPS) |
| PD-L1+ cutoff | CPS≥10 | IC≥1% | IC≥1% | IC≥1% | CPS≥1 |
| ICI agent | Pembrolizumab | Atezolizumab | Atezolizumab | Atezolizumab | Toripalimab |
| Chemotherapy | Investigator choice | nab-PTX | PTX | Gem/Cb or Cape | nab-PTX |
| Steroid premed | Per regimen | No | Yes (dex) | Per regimen | No |
| Final FU (mo) | ~44 | ~19 | ~24 | ~15 | ~26 |
| Key result | Positive | ITT- OS+ | Negative | Negative | Positive |

### Early-Stage Setting

| Characteristic | KEYNOTE-522 | IMpassion031 | GeparNuevo | NeoTRIP | A-BRAVE |
| --- | --- | --- | --- | --- | --- |
| Phase | III | III | II | III | III |
| Blinding | Double-blind | Double-blind | Double-blind | Open-label | Open-label |
| NCT | NCT03036488 | NCT03197935 | NCT02685059 | NCT02620280 | NCT02926196 |
| N | 1,174 | 333 | 174 | 280 | 466 |
| Countries | 21 | 13 | Germany | 5 | Europe |
| Median Age (yr) | 49 | 50 | 49 | 50 | 51 |
| Stage II/III (%) | 25/75 | ~23/77 | ~35/65 | ~30/70 | Post-op |
| Node-positive (%) | ~52 | ~45 | ~40 | ~55 | ~65 |
| PD-L1 assay | 22C3 (CPS) | SP142 (IC) | SP263 | SP142 (IC) | 73-10 |
| ICI agent | Pembrolizumab | Atezolizumab | Durvalumab | Atezolizumab | Avelumab |
| Neoadjuvant chemo | Cb/PTX → AC | nab-PTX → AC | nab-PTX → EC | Cb/nab-PTX | — |
| Adjuvant ICI | Yes (27 weeks) | Yes (~24 weeks) | No | No (anthracycline) | Yes (1 year) |
| Carboplatin | Yes | No | No | Yes | — |
| Final FU (mo) | ~75 | ~40 | ~86 (7 yr) | ~54 | ~52 |
| Key result | Positive | Positive | Positive (Ph II) | Negative | OS+ / DFS- |

**Abbreviations:** Cb=carboplatin; PTX=paclitaxel; nab-PTX=nab-paclitaxel; AC=doxorubicin/cyclophosphamide; EC=epirubicin/cyclophosphamide; Cape=capecitabine; Gem=gemcitabine; FU=follow-up; dex=dexamethasone; ITT- OS+=ITT OS not significant, PD-L1+ OS significant; OS+/DFS-=OS significant, DFS not significant.

## Supplementary Table 3. Fixed-Effect vs Random-Effects Model Comparison

| Outcome | Fixed-Effect HR/OR (95% CI) | Random-Effects HR/OR (95% CI) |
| --- | --- | --- |
| Metastatic OS ITT | 0.85 (0.76-0.94) | 0.85 (0.74-0.96) |
| Metastatic OS PD-L1+ | 0.78 (0.69-0.89) | 0.79 (0.65-0.95) |
| Metastatic PFS ITT | 0.82 (0.75-0.90) | 0.82 (0.73-0.92) |
| Early EFS/iDFS | 0.74 (0.64-0.86) | 0.75 (0.63-0.90) |
| Early OS | 0.64 (0.53-0.78) | 0.64 (0.53-0.78) |
| pCR (OR) | 1.66 (1.39-1.97) | 1.65 (1.39-1.98) |

## Supplementary Table 4. GRADE Certainty of Evidence Summary

| Outcome | Study Design | Risk of Bias | Inconsistency | Indirectness | Imprecision | Other | Certainty |
| --- | --- | --- | --- | --- | --- | --- | --- |
| Metastatic OS (ITT) | 5 RCTs | Not serious | Not serious (I²=27%) | Not serious | Serious¹ | None | MODERATE |
| Metastatic OS (PD-L1+) | 5 RCTs | Not serious | Serious (I²=51%) | Serious² | Not serious | None | LOW |
| Metastatic PFS (ITT) | 5 RCTs | Not serious | Not serious (I²=35%) | Not serious | Not serious | None | MODERATE |
| Early EFS/iDFS | 5 RCTs | Not serious | Not serious (I²=0.2%) | Serious³ | Not serious | None | MODERATE |
| Early OS | 4 RCTs | Not serious | Not serious (I²=0%) | Not serious | Serious⁴ | None | MODERATE |
| pCR | 4 RCTs | Not serious | Not serious (I²=1%) | Not serious | Not serious | None | HIGH |

¹ HKSJ-adjusted CI crosses 1.0 (0.70-1.03, P=0.076).
² PD-L1 positivity defined by non-equivalent assays and thresholds across trials.
³ Pools EFS, iDFS, and DFS endpoints; combines neoadjuvant and adjuvant strategies.
⁴ Dominated by KEYNOTE-522; exclusion of this trial broadens CI substantially.

## Supplementary Table 5. Trial-Level Data Extraction Dataset

Input data used for all meta-analyses. All HRs, ORs, and CIs verified by two reviewers (B.G., R.Z.) against source publications.

### Metastatic Setting

| Trial | OS HR | OS 95% CI | PFS HR | PFS 95% CI | Source |
| --- | --- | --- | --- | --- | --- |
| KEYNOTE-355 | 0.73 | (0.55-0.95) | 0.65 | (0.49-0.86) | [22] (CPS>=10) |
| IMpassion130 | 0.87 | (0.75-1.02) | 0.80 | (0.69-0.92) | [34] (final OS), [7] (PFS) |
| IMpassion131 | 1.11 | (0.76-1.64) | 0.86 | (0.70-1.06) | [8] |
| IMpassion132 | 0.93 | (0.73-1.20) | 0.98 | (0.80-1.20) | [37] |
| TORCHLIGHT | 0.69 | (0.51-0.93) | 0.77 | (0.60-0.99) | [23] |

### Early-Stage Setting

| Trial | EFS HR | EFS 95% CI | OS HR | OS 95% CI | pCR % (Exp/ Ctrl) | Source |
| --- | --- | --- | --- | --- | --- | --- |
| KEYNOTE-522 | 0.65 | (0.51-0.83) | 0.66 | (0.50-0.87) | 64.8/51.2 | [11] |
| IMpassion031 | 0.76 | (0.54-1.07) | 0.69 | (0.42-1.13) | 57.6/41.1 | [12] |
| GeparNuevo | 0.56 | (0.32-0.99) | 0.33 | (0.14-0.79) | 53.4/44.2 | [35] (iDFS/OS), [13] (pCR) |
| NeoTRIP | 1.08 | (0.70-1.67) | — | — | 48.6/44.4 | [36] (EFS), [14] (pCR) |
| A-BRAVE | 0.81 | (0.61-1.09) | 0.66 | (0.45-0.97) | — | [15] (DFS/OS) |

## Supplementary Table 6. Population Definitions for Pooled Analyses

| Pooled Analysis | Trials | Population | Patients Contributing |
| --- | --- | --- | --- |
| Metastatic OS ITT | 5 | ITT (all-comers) for KEYNOTE-355, IMpassion130, IMpassion131; PD-L1-enriched for IMpassion132; all-comers for TORCHLIGHT | 3,526 |
| Metastatic OS PD-L1+ | 5 | Trial-defined PD-L1+ primary population: CPS>=10 (KM-355), IC>=1% (IM130/131/132), CPS>=1 (TORCHLIGHT) | ~2,100 |
| Metastatic PFS ITT | 5 | Same populations as OS ITT | 3,526 |
| Early EFS/iDFS | 5 | ITT for all 5 trials (EFS for KN-522, IM031, NeoTRIP; iDFS for GeparNuevo; DFS for A-BRAVE) | 2,427 |
| Early OS | 4 | ITT (KEYNOTE-522, IMpassion031, GeparNuevo, A-BRAVE) | ~2,147 |
| pCR | 4 | ITT with evaluable surgical specimens (KEYNOTE-522, IMpassion031, GeparNuevo, NeoTRIP) | 1,961 |
| Safety (irAEs) | 10 | As-treated/safety population from all 10 trials | ~5,900 |
| Safety (grade >=3 TRAEs) | 10 | As-treated/safety population from all 10 trials | ~5,900 |

## Supplementary Table 7. Leave-One-Out Sensitivity Analysis of Anti-PD-1 vs Anti-PD-L1 Subgroup Comparison (Metastatic OS)

| Sensitivity scenario | anti-PD-1 HR (95% CI) | anti-PD-L1 HR (95% CI) | Subgroup P |
| --- | --- | --- | --- |
| Full analysis (5 trials) | 0.71 (0.58-0.87) | 0.91 (0.80-1.03) | 0.045 |
| Excl. IMpassion131 (negative trial) | 0.71 (0.58-0.87) | 0.89 (0.78-1.01) | 0.074 |
| Excl. IMpassion132 (early-relapse) | 0.71 (0.58-0.87) | 0.92 (0.75-1.12) | 0.077 |
| Excl. TORCHLIGHT (Chinese only) | 0.73 (0.56-0.96) | 0.91 (0.80-1.03) | 0.156 |
| Excl. KEYNOTE-355 | 0.69 (0.51-0.93) | 0.91 (0.80-1.03) | 0.099 |
| Backbone-matched (nab-paclitaxel only) | 0.69 (0.51-0.93) | 0.87 (0.75-1.01) | 0.178 |

**Interpretation:** The nominal anti-PD-1 versus anti-PD-L1 subgroup difference (P=0.045) was not robust: exclusion of any single trial rendered the difference non-significant (P=0.074-0.156), and the backbone-matched comparison showed no significant difference (P=0.178). This indicates the apparent difference is trial-driven rather than mechanism-driven; no superiority of PD-1 over PD-L1 blockade should be inferred.

*Note: Supplementary Figures 1A-D (funnel plots) and Supplementary Figure 2 (leave-one-out sensitivity analysis) are provided as separate image files.*
